# Robust optimization of convolutional neural networks with a uniform experiment design method: a case of phonocardiogram testing in patients with heart diseases

**DOI:** 10.1186/s12859-021-04032-8

**Published:** 2021-11-08

**Authors:** Wen-Hsien Ho, Tian-Hsiang Huang, Po-Yuan Yang, Jyh-Horng Chou, Jin-Yi Qu, Po-Chih Chang, Fu-I. Chou, Jinn-Tsong Tsai

**Affiliations:** 1 Department of Healthcare Administration and Medical Informatics, Kaohsiung Medical University, No.100, Shin-Chuan 1st Road, Kaohsiung, 807 Taiwan; 2 Department of Medical Research, Kaohsiung Medical University Hospital, No.100, Shin-Chuan 1st Road, Kaohsiung, 807 Taiwan; 3 Center for Big Data Research, Kaohsiung Medical University, No.100, Shin-Chuan 1st Road, Kaohsiung, 807 Taiwan; 4 Department of Information Engineering and Computer Science, Feng Chia University, No. 100, Wenhwa Road, Taichung, 407 Taiwan; 5 Department of Mechanical Engineering, National Chung-Hsing University, No. 145, Xingda Road, Taichung, 402 Taiwan; 6 Department of Electrical Engineering, National Kaohsiung University of Science and Technology, No. 415, Chien-Kung Road, Kaohsiung, 807 Taiwan; 7 Division of Thoracic Surgery, Department of Surgery, Kaohsiung Medical University Hospital, No.100, Shin-Chuan 1st Road, Kaohsiung, 807 Taiwan; 8 Weight Management Center, Kaohsiung Medical University Hospital, No.100, Shin-Chuan 1st Road, Kaohsiung, 807 Taiwan; 9 College of Medicine, Ph.D. Program in Biomedical Engineering, Kaohsiung Medical University, No.100, Shin-Chuan 1st Road, Kaohsiung, 807 Taiwan; 10 Department of Sports Medicine, College of Medicine, Kaohsiung Medical University, No.100, Shin-Chuan 1st Road, Kaohsiung, 807 Taiwan; 11 Department of Computer Science, National Pingtung University, No. 4-18, Min-Sheng Road, Pingtung, 900 Taiwan

**Keywords:** Phonocardiogram, Heart disease, Robust optimization, Convolutional neural network, Uniform design

## Abstract

**Background:**

Heart sound measurement is crucial for analyzing and diagnosing patients with heart diseases. This study employed phonocardiogram signals as the input signal for heart disease analysis due to the accessibility of the respective method. This study referenced preprocessing techniques proposed by other researchers for the conversion of phonocardiogram signals into characteristic images composed using frequency subband. Image recognition was then conducted through the use of convolutional neural networks (CNNs), in order to classify the predicted of phonocardiogram signals as normal or abnormal. However, CNN requires the tuning of multiple hyperparameters, which entails an optimization problem for the hyperparameters in the model. To maximize CNN robustness, the uniform experiment design method and a science-based methodical experiment design were used to optimize CNN hyperparameters in this study.

**Results:**

An artificial intelligence prediction model was constructed using CNN, and the uniform experiment design method was proposed to acquire hyperparameters for optimal CNN robustness. The results indicate Filters ($${X}_{1}$$), Stride ($${X}_{3}$$), Activation functions ($${X}_{6}$$), and Dropout ($${X}_{7}$$) to be significant factors considerably influencing the ability of CNN to distinguish among heart sound states. Finally, the confirmation experiment was conducted, and the hyperparameter combination for optimal model robustness was Filters ($${X}_{1}$$) = 32, Kernel Size ($${X}_{2})$$ = 3 × 3, Stride ($${X}_{3}$$) = (1,1), Padding ($${X}_{4})$$ as same, Optimizer ($${X}_{5})$$ as the stochastic gradient descent, Activation functions ($${X}_{6}$$) as relu, and Dropout ($${X}_{7}$$) = 0.544. With this combination of parameters, the model had an average prediction accuracy rate of 0.787 and standard deviation of 0.

**Conclusion:**

In this study, phonocardiogram signals were used for the early prediction of heart diseases. The science-based and methodical uniform experiment design was used for the optimization of CNN hyperparameters to construct a CNN with optimal robustness. The results revealed that the constructed model exhibited robustness and an acceptable accuracy rate. Other literature has failed to address hyperparameter optimization problems in CNN; a method is subsequently proposed for robust CNN optimization, thereby solving this problem.

## Background

Phonocardiogram (PCG) and electrocardiograph (ECG) signals are commonly used for observing and analyzing heart diseases. Vibration waves generated by the turbulent blood flow, the contraction of the myocardium, the closing of the heart valves, and the vibrations generated by the blood impact on ventricular or aortic walls create PCG signals [1]. Adults with healthy hearts produce two distinctive heart sounds per cardiac cycle, namely S1 and S2. Other sounds may also occur during the cardiac cycle, such as S3, S4, and heart murmurs. Heart sound intensity, heart sound frequency, and the relationship between each heart sound reflect the condition of the heart valve, cardiac muscle function, and blood flow inside the heart. Using stethoscopes, physicians can hear patients’ heartbeats and observe changes in heart sounds to determine their heart disease condition [1]. Accordingly, PCG signals are vital to the analysis and diagnosis of heart diseases. Because PCG signals are easier to acquire than ECG signals, this study employed PCG signals as the input signals for heart disease analysis.

Scholars have used the Markov model to classify heart sounds in the cardiac cycle; some have included heart sound duration and the variations among heart sound states in their analyses. To distinguish S1 and S2, Schmidt et al. [1] combined the duration of heart sounds collected in clinical environments with the duration-dependent hidden Markov model to classify heart sound state, yielding a 98.8% accuracy rate. Springer et al. [2] integrated the use of logistic regression–based hidden semi-Markov model and heart sound duration, achieving an average F_1_ score of 95.63 ± 0.85%. Liu et al. [3] used eight public datasets to evaluate the performance of the logistic regression-based hidden semi-Markov model in distinguishing S1 and silent systole states as well as S2 and silent diastole states, revealing an average F_1_ score of 98.5% and 97.2%, respectively.

In heart sound collection, environmental influences may cause excessive noise in PCG signals, creating problems in subsequent identification and analysis. Therefore, noise preprocessing is necessary for the effective extraction and computation of signal characteristics in heart sound data and for the enhancement of model accuracy. Various denoising techniques, such as the wavelet packet transform technique, are available for reducing noise in sound. Messer et al. [4] employed optimal wavelet packet transform to successfully reduce noise in the PCG signals of patients with heart diseases and analyzed optimal wavelet families, decomposition criteria, and threshold values for noise reduction in heart sound data. Scully et al. [5] used the wavelet packet transform technique and verified that biological signal parameters detectable by mobile phones, including breathing frequency, cardiac R–R intervals, and blood oxygen saturation, are accurate enough for analysis. Joy et al. [6] proposed a wavelet packet transformation technique that uses a simple threshold rule to stably increase the signal-to-noise ratio (SNR). Zeng et al. [7] combined the fast Fourier transform technique with the wavelet packet transform technique to reduce noise in PCG signals collected with wearable electronic medical devices; their results revealed that the method effectively filtered out PCG signal noise and successfully retained pathology information.

Because the PCG signal discussed in this study is within a certain frequency range, in addition to using the wavelet packet transformation technique for signal preprocessing, this study employed wave filtering preprocessing to achieve the required preprocessing effect. This method has been used by other scholars to process PCG signals. For example, Potes et al. [8] passed PCG signals through a band pass filter to control the signal frequency between 25 and 400 Hz and conducted subsequent analysis; Bozkurt et al. [9] used the gammatone filter for PCG signal preprocessing. The two aforementioned study cases indicate that wave-processed PCG signals improve subsequent analysis. Therefore, this study adopted a feature extraction approach, extracting PCG signals within a specific range, to effectively mitigate the influence of PCG signal noise.

Deep-learning techniques have been widely applied in intelligent classification. For example, convolutional neural network (CNNs), a common deep-learning technique in which data is processed before use, serves as the basis for heart sound classification [8–10]. Based on the three aforementioned studies, CNN-based heart sound classification techniques yield favorable outcomes. Therefore, this study employed CNNs as the core system for heart sound classification.

However, CNNs require the tuning of multiple hyperparameters, resulting in the hyperparameter optimization problem. To construct a robust and optimized CNN, this study adopted the uniform design experiment approach and used the science-based and methodical experiment design method to optimize CNN hyperparameters.

## Methods

### Data preprocessing

**Fig. 1:**
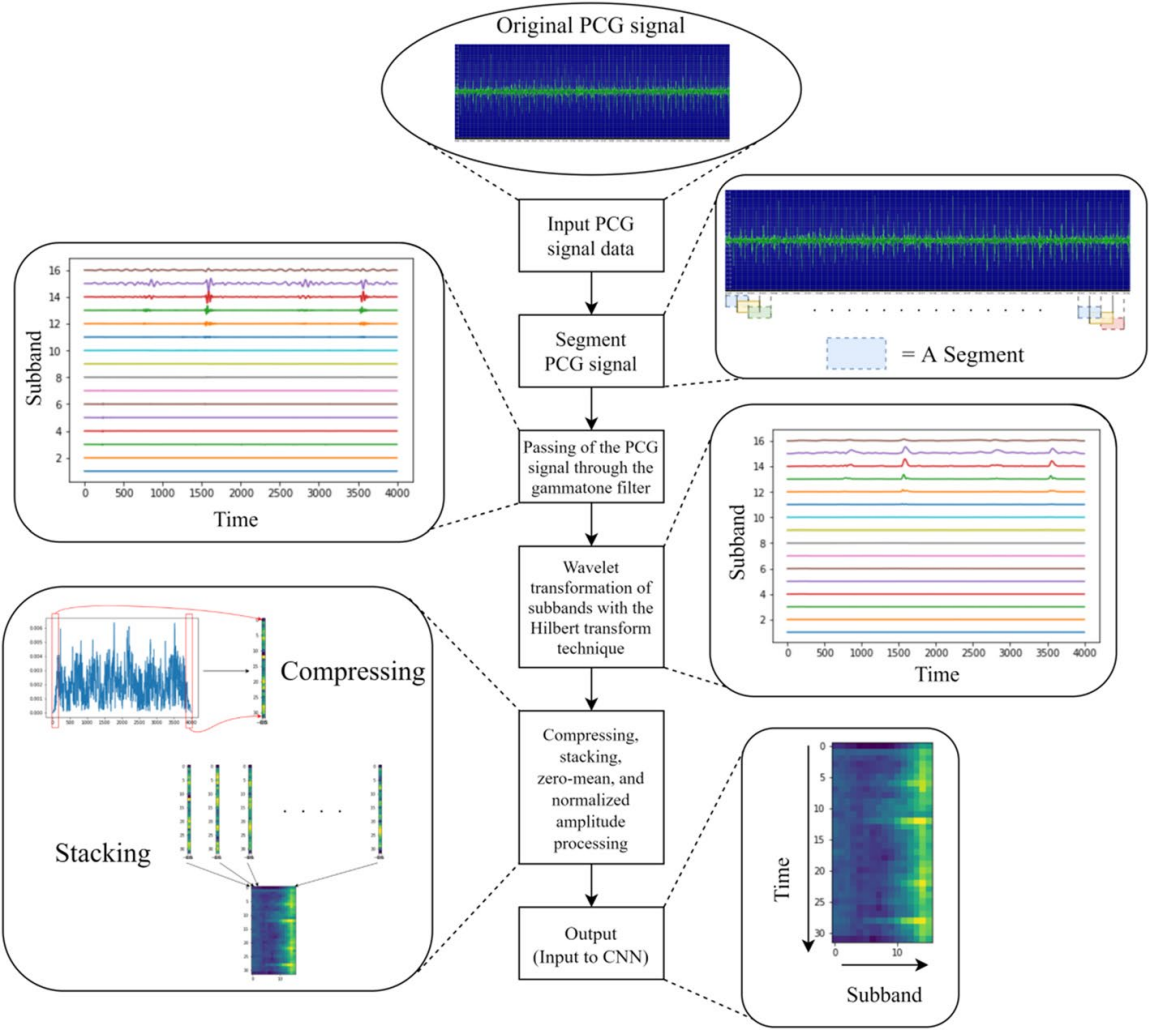
Data preprocessing process

Figure 1 presents the preprocessing process, in which PCG signals are segmented using asynchronous methods [9]; this requires the setting of two parameters. The frame size is set as 2 s and the hop size as 1 s. Subsequently, the gammatone filter is employed as the wave filter to further segment the segmented PCG signal. The computation process is as follows:

Step 1: Based on the set frequency bands, compute the central frequency for the corresponding counts of data [11].
Step 2: Use the central frequency to compute 10 wave filter parameters for each band, which are used in the four independent linear wave filters.
Step 3: According to their serial numbers, input segmented PCG signals into the wave filters with set parameters for computation. The filtered results are the subband signals.

In this study, the frequency band was set to contain 16 subbands, resulting in central frequencies of 878.06, 768.221, 669.281, 580.159, 499.881, 427.569, 362.432, 303.76, 250.909, 203.304, 160.422, 121.795, 87.0014, 55.6604, 27.4295, and 2 Hz, which computed by Python codes [11].

Subsequently, the Hilbert transform technique was applied for the wavelet transformation of subbands. After numerous conversions, the real-valued signals were converted into complex signals comprising complex numbers. Subsequently, the absolute values of the numbers were extracted to generate the wavelet effect of the signals.

The data were then compressed to the set time resolutions (32 in this study) and subjected to zero-mean and normalized amplitude processing. Standardized data processing facilitates and enhances the convergence speed and performance of the subsequent classification model.

### Convolution neural network hyperparameters

CNN comprises three main layers, namely the convolutional, pooling, and fully connected layer. In order to compare with the best system developed by Bozkurt et al. [9], the CNN model used in this study includes 2 convolutional layers followed by max-pooling and drop-out layers. In this study, the following considered but not limited hyperparameters require tuning in the convolutional layer: “Filters,” “Kernel Size,” “Stride,” “Padding,” and “Activation function.” In the fully connected layer, only the Dropout percentage hyperparameter, “Dropout,” requires tuning. Therefore, Python software language was used to construct the system. When Python is used to train CNN by using the Keras module, the “Optimizers” hyperparameter must be set. Various types of optimizers are available for use, including the stochastic gradient descent and the Adam optimizer.

In summary, the optimization of multiple hyperparameters is necessary for CNN construction. Accordingly, this study employed the experimental design method to optimize the classification model.

### Use of an experiment design method to stabilize and optimize the classification model

The experiment design method employs mathematical statistics and uses methodical science-based procedures and methods to design suitable experiments. The method thereby reduces the number of times the experiment must be repeated, reducing the time and money required, and optimizes the experiment process through suitable analysis methods. This study employed the uniform design method as its experiment design method [12–15]. A key feature in uniform design is the uniform layout distribution of the factor levels in an experiment, which results in experiment points that are uniformly scattered within the range of the experiment parameters. Because it leads to experiments in which the experiment points are uniformly scattered, uniform design is suitable for optimization with fewer repeated experiments under high level numbers and wide parameter ranges. Accordingly, it is suitable for solving and searching optimization problems on a global scale.

In contrast with the uniform design method, another famous experiment design method named “Taguchi method” is suitable for fewer factors, fewer factor levels and factors with interaction. Generally speaking, Taguchi method was adopted to solving and searching optimization problems on a local area due to fewer factor levels. Therefore, Taguchi method is not appropriate for this study.

The results of uniform design experiments enable researchers to identify the optimal combination of variables in the experiment through direct observation. Researchers can also use regression analysis to compute parameter model regression equations. By using this regression equation as the objective function, researchers can employ genetic algorithms to compute the optimal combination of parameters and directly search for the optimal combination within the limited number range [16–18].

### Model evaluation method

After model training was complete, the researchers input the test set into the model to evaluate its performance. The computed results were first compiled into a confusion matrix, and the assessment indicators, namely accuracy, F-score, sensitivity, and specificity, were separately computed [19].

The values of the suitable evaluation indicators were converted using the SNR to facilitate the analysis of the experiment design results. The SNR equation for each experiment is as follows:

1 $${\mathrm{SNR}}=-10log\left[{\left( \bar{x} -m\right)}^{2}+{\sigma }^{2}\right],$$

in which $\stackrel{-}{x}$ is the average value of the evaluation indicator results ($\bar{x}=\sum_{i=1}^{n}\frac{{x}_{i}}{n})$, *m* is the target value (*m* = 1),σ is the standard deviation ($\sigma =\sqrt{\frac{1}{n-1}\sum_{i=1}^{n}{\left({x}_{i}- \bar{x}\right)}^{2}})$ and *n* is the repeated times for each experiment.

## Results and discussion

### Description of datasets

In this study, data were collected from the PCG signal database PhysioNet/CinC Challenge 2016 [20]. Because the datasets were unbalanced, data augmentation was necessary in the training set to increase the counts of abnormal PCG signals and balance the data counts for normal and abnormal data [9]. For data augmentation, the researchers conducted upsampling on randomly-selected cases of abnormal PCG signal data. After the amplitudes of the selected PCG signals are randomly adjusted from 10 to 20%, the adjusted signals are saved as a new PCG signal file. Once the counts are equal for normal and abnormal data, the data expansion procedure is complete. The distribution of normal and abnormal PCG data in the training set, validation set, and test set are presented in Table 1.

**Table 1:** Distribution of PCG signal data

Dataset	Normal	Abnormal	Total
Training set	1854	1854	3708
Validation set	502	502	1004
Test set	150	151	301

### Results and discussion of uniform design experiments

As described, the construction of CNNs requires the optimization of multiple hyperparameters. In this study, seven hyperparameters were selected for the experiment design, namely Filters ($${X}_{1})$$, Kernel Size ($${X}_{2})$$, Stride ($${X}_{3})$$, Padding ($${X}_{4})$$, Optimizer ($${X}_{5})$$, Activation functions ($${X}_{6})$$, and Dropout ($${X}_{7})$$. The experiment design was planned using the U_10_(10^7^) Uniform Experiment Design Table (Table 2). Note that the learning rates of Optimizer SGD and Adam are setting as 0.01 and 0.001, respectively.

**Table 2:** U_10_(10^7^) Uniform experiment design

Experiment number	Filters	Kernel size	Stride	Padding	Optimizer	Activation functions	Dropout
1	2	3 × 3	1 × 1	Valid	SGD	relu	0.8
2	4	3 × 3	2 × 2	Valid	Adam	relu	0.6
3	8	3 × 3	1 × 1	Same	Adam	tanh	0.4
4	16	3 × 3	1 × 1	Same	SGD	tanh	0.2
5	32	3 × 3	2 × 2	Same	SGD	tanh	0
6	2	2 × 2	1 × 1	Valid	Adam	relu	0.8
7	4	2 × 2	2 × 2	Valid	Adam	relu	0.6
8	8	2 × 2	2 × 2	Valid	SGD	relu	0.4
9	16	2 × 2	1 × 1	Same	SGD	tanh	0.2
10	32	2 × 2	2 × 2	Same	Adam	tanh	0

According to the experiment distribution in Table 2, the researchers executed 10 sets of experiments and repeated each experiment five times (${x}_{i}, i=1, 2,\dots , 5)$. The researchers used Eq. (1) to convert the accuracy parameter values into the SNR values. Table 3 presents the SNR evaluation results of the training, validation, and test set. The results indicate that the combinations in experiments 4 and 9 may be optimal; both experiments yield high SNR values, namely − 0.9971 and − 0.7069, respectively.

**Table 3:** SNR evaluation results for the training, validation, and test sets

Experiment combination	Training set SNR	Validation set SNR	Test set SNR
1	− 8.8592	− 5.2119	− 4.7965
2	− 10.9555	− 7.7709	− 4.4645
3	− 3.2841	− 1.9047	− 1.9789
4	− 0.1185	− 0.0403	− 0.9971
5	− 2.7631	− 1.4347	− 3.7911
6	− 8.2711	− 5.8263	− 4.4475
7	− 10.3979	− 8.3514	− 5.2433
8	− 6.9688	− 4.5524	− 4.2700
9	− 0.1878	− 0.6176	− 0.7069
10	− 2.4236	− 2.1116	− 2.9854

By using data from Table 3 as regression analysis data, the researchers used the test set SNR as the dependent variable ($$Y$$) and Filters ($${X}_{1})$$, Kernel Size ($${X}_{2})$$, Stride ($${X}_{3})$$, Padding ($${X}_{4})$$, Optimizer ($${X}_{5})$$, Activation functions ($${X}_{6})$$, and Dropout ($${X}_{7})$$ as independent variables. The regression equation is presented in Eq. (2) (R-value = 0.9997):

2 $$Y=\left(-22.783\right)+34.58{X}_{1}+\left(-8.5734\right){X}_{3}+\left(-5.4072\right){X}_{6}+58.978{X}_{7}+(-43.237){X}_{7}^{2}$$

In the equation, the *p* values of Filters ($${X}_{1}$$), Stride ($${X}_{3}$$), Activation functions ($${X}_{6}$$), and Dropout ($${X}_{7}$$) are < 0.05, indicating that these factors are significant and affect the model’s performance more. Genetic algorithm searching for the optimal parameter combination by using Eq. (2) revealed the optimal combination to be Filters ($${X}_{1}$$) = 32, Stride ($${X}_{3}$$) = (1,1), Activation functions ($${X}_{6}$$) as relu, and Dropout ($${X}_{7}$$) = 0.544. The experiment results presented in Tables 2 and 3 reveal that the model’s performance improved when the Optimizer ($${X}_{5})$$ parameter was set as the SGD. The optimal values for Kernel Size ($${X}_{2})$$ and Padding ($${X}_{4})$$ are obtained in subsequent experiments.

### Validation experiment results and discussion

The researchers next conducted full factorial experiments, namely confirmation experiments, on the nonsignificant factors Kernel Size ($${X}_{2})$$ and Padding ($${X}_{4})$$; each set of experiments was repeated three times. Table 4 presents the experiment combinations of the confirmation experiment and Table 5 presents the test accuracy rate, F_1_ score and False Positive. The results indicate that, when the significant factors are set, the model retains robustness, and no major changes are exhibited when other factors are adjusted. Therefore, Kernel Size ($${X}_{2})$$ and Padding ($${X}_{4})$$ are nonsignificant factors. Table 5 indicates that the combination in Experiment 3 had the highest mean accuracy rate, 0.787, and a standard deviation of closing to 0 as well as highest F1. Therefore, the hyperparameter combination that optimized the model’s robustness is confirmed to be Filters ($${X}_{1}$$) = 32, Kernel Size ($${X}_{2})$$ = 3 × 3, Stride ($${X}_{3}$$) = (1,1), Padding ($${X}_{4})$$ as same, Optimizer ($${X}_{5})$$ as the SGD, Activation functions ($${X}_{6}$$) as relu, and Dropout ($${X}_{7}$$) = 0.544. As a result of the comparison with same test data, the combination in Experiment 3 finally obtained the prediction effect with the accuracy of 0.951, sensitivity of 0.892 and specificity of 0.953, which is better than the best system developed by Bozkurt et al. [9] with the accuracy of 0.815, sensitivity of 0.815 and specificity of 0.785.

**Table 4:** Experiment combinations of the confirmation experiment for optimal Kernel Size (*X*_2_) and Padding (*X*_4_)

Experiment combination	Filters	Kernel size	Stride	Padding	Optimizer	Activation functions	Dropout
1	32	2 × 2	1 × 1	Same	SGD	relu	0.544
2	32	2 × 2	1 × 1	Valid	SGD	relu	0.544
3	32	3 × 3	1 × 1	Same	SGD	relu	0.544
4	32	3 × 3	1 × 1	Valid	SGD	relu	0.544

**Table 5:** Experimental results of the experiment combinations in Table 4

Experiment combination	Accuracy		F_1_		False positive	
	Mean	SD	Mean	SD	Mean	SD
1	0.753	0.02	0.772	0.01	0.168	0.04
2	0.766	0.019	0.782	0.019	0.166	0.035
3	0.787	≈ 0	0.793	0.002	0.19	0.01
4	0.786	0.015	0.792	0.011	0.188	0.008

## Conclusions

To achieve the early prediction of heart diseases, this study employed PCG signals for heart disease analysis and CNN for the construction of an artificial intelligence prediction model. After data preprocessing, the uniform experiment design method was adopted to derive hyperparameters yielding a CNN with optimal robustness. The results revealed Filters ($${X}_{1}$$), Stride ($${X}_{3}$$), Activation functions ($${X}_{6}$$), and Dropout ($${X}_{7}$$) to be significant factors, each of which considerably influences the discrimination ability of CNN. Finally, the confirmation experiment revealed the hyperparameter combination that optimized the model’s robustness: Filters ($${X}_{1}$$) = 32, Kernel Size ($${X}_{2})$$= 3 × 3, Stride ($${X}_{3}$$) = (1,1), Padding ($${X}_{4})$$ as same, Optimizer ($${X}_{5})$$ as SGD, Activation functions ($${X}_{6}$$) as relu, and Dropout ($${X}_{7}$$) = 0.544. This combination provided the model with mean testing accuracy of 0.787 and a standard deviation of closing to 0. The results reveal that this combination of parameters yields a model with a high level of robustness and acceptable accuracy. Other literature has failed to address the CNN hyperparameter optimization problem, so this study proposes a solution for robust optimization of the model.

## Data Availability

The datasets analysed during the current study are available in the Classification of Heart Sound Recordings—the PhysioNet Computing in Cardiology Challenge 2016, https://physionet.org/content/challenge-2016/1.0.0/.

## Abbreviations

CNN: Convolutional neural networks
PCG: Phonocardiogram
ECG: Electrocardiograph
SNR: Signal-to-noise ratio
